# A Meta-Analysis of Circulating Microvesicles in Patients with Myocardial Infarction

**DOI:** 10.5935/abc.20170102

**Published:** 2017-08

**Authors:** Zhida Wang, Wang Cai, Shaolan Hu, Yufei Xia, Yao Wang, Qi Zhang, Liming Chen

**Affiliations:** 1 Key Laboratory of Hormones and Development (Ministry of Health) - Tianjin Key Laboratory of Metabolic Diseases - Tianjin Metabolic Diseases Hospital & Tianjin Institute of Endocrinology - Tianjin Medical University, Tianjin - China; 2 Department of Surgery - Tianjin Nankai Hospital - Tianjin Medical University, Tianjin, China; 3 School of Nursing - Tianjin Medical University, Tianjin - China; 4 Department of Pharmacology - School of Basic Medical Science - Tianjin Medical University, Tianjin - China; 5 Institute of Integrative Medicines for Acute Abdominal Diseases - Nankai Hospital, Tianjin - China

**Keywords:** Myocardiaol Infarction, Biomarkers, Cell-Derived Microparticles, Annexion A5, Blood Platelets, Leukocytes, Endothelium

## Abstract

**Background:**

Cell-derived microvesicles (MVs) are vesicles released from activated or apoptotic cells. However, the levels of MVs in myocardial infarction have been found inconsistent in researches.

**Objective:**

To assess the association between MVs and myocardial infarction by conducting a meta-analysis.

**Methods:**

A systematic literature search on PubMed, Embase, Cochran, Google Scholar electronic database was conducted. Comparison of the MVs levels between myocardial infarction patients and healthy persons were included in our study. Standard Mean Difference (SMD) and 95% confidence interval (CI) in groups were calculated and meta-analyzed.

**Results:**

11 studies with a total of 436 participants were included. Compared with the health persons, AMVs [SMD = 3.65, 95% CI (1.03, 6.27)], PMVs [SMD = 2.88, 95% CI (1.82, 3.93),] and EMVs [SMD = 2.73, 95% CI (1.13, 4.34)], levels were higher in patients with myocardial infarction. However, LMVs levels [SMD = 0.73, 95% CI (-0.57, 2.03)] were not changed significantly in patients with myocardial infarction.

**Conclusions:**

AMVs, PMVs and EMVs might be potential biomarkers for myocardial infarction.

## Introduction

Ischemic Heart Disease (IHD) is one of the cardiovascular diseases, which impairs human health.^1^ Atherothrombosis, endothelial dysfunction and cell apoptosis are on the pathologic basis in these diseases. The relevant studies in this area have suggested that cell-derived microvesicles (MVs) are related with platelet activation, endothelial damage and inflammation associated with the existence of cardiovascular risk factors.^2-4^ Since they are involved in the pathophysiologic process of diseases, attention has being focused on the relationship between MVs and myocardial infarction (MI).^5-8^ Increasing evidences imply that MVs might be considered as novel biomarkers or mediators helpful in understanding the mechanisms of cardiovascular diseases.

MVs are used to describe a population of sub-cellular vesicles released from plasma membrane during cell activation or apoptosis and identified by size range from 100 nm to 1.0 µm in diameter. MVs constitute a heterogeneous population, different in cellular origin, numbers, size, antigenic composition, and functional properties. Alterations in the amounts of different cell-derived MVs may provide information on the pathophysiologic changes. Although many studies have shown that myocardial infarction is associated with MVs, the information obtained shows heterogeneous result, with a high variation regarding MVs size, MVs type, MVs levels, inclusion criteria and methods. Thus, we performed a meta-analysis of the changes of Annexin V positive MVs (AMVs), platelet MVs (PMVs), endothelial MVs (EMVs) and leukocytes MVs (LMVs) in patients with myocardial infarction and healthy persons.

## Methods

### Data Sources and Searches

We searched the databases of MEDLINE (pubmed), Embase, Cochrane and Google Scholar electronic database for articles from 2000 to 2013. All searches were applied with the following medical subject headings: “myocardial infarction”, “primary percutaneous coronary intervention”, “stenting”, “balloon angioplasty”, “acute coronary syndrome” or “coronary ischemia”, “microparticles”, “cell-derived microparticles”, “circulating microparticles”, “microvesicles”. These searches were restricted to publications limited to research on humans. A manual search for references cited in the published studies and relevant review articles was also performed to identify additionally suitable investigations for our purpose. For unpublished and published studies that were not exhaustively disclosed, the attempt through e-mail was made to contact principal investigators in order to retrieve missing data. Finally, well-known experts in this area were contacted to ensure that all relevant data were captured.

### Study Selection

Two of us performed the identification of relevant abstracts and the selection of studies based on the criteria described below independently, and a third investigator resolved any discrepancy. We selected studies comparing the levels of diverse MVs: total, platelet-, endothelial-, leukocyte-derived between healthy persons and patients with myocardial infarction.

Studies were included if they met the following criteria: (i) Study entitled circulating MVs correlated with MI; (ii) Design of study was case-control study or cohort study; (iii) The MI as a research subgroup independently extract relevant information MI. We excluded the following: (i) Review; (ii) Not full text, only a summary; (iii) Animal testing.

### Data Extraction and Synthesis

We extracted information including study and population characteristics, sample size, study design, and outcomes relevant to this study. Means and standard deviations of MVs levels were extracted. When an article complied with the inclusion criteria but lacked information on parameters for analysis, or when outcomes were reported but not related to myocardial infarction, we contacted the authors to obtain raw data. The quality of studies was assessed using the Downs and Black checklist.

### Statistical Analysis

The data was analyzed using RevMan 5.0 statistical software provided by the Cochrane Collaboration analyzed. SMD and 95% CI were used as summary estimates. The presence of heterogeneity between studies was tested with the χ² test for heterogeneity and the I² statistic. Heterogeneity was significant when p< 0.05 or I² was more than 50%. A random-effects model was used in all analyses to test the stability of the results to the choice of the statistical model. If significant heterogeneity, results of the random-effects model are used. We defined a priori sensitivity analysis of high-quality studies for each clinical outcome. The potential for publication bias was evaluated using the funnel plot approach.

## Results

### Search Results

**Figure 1:**
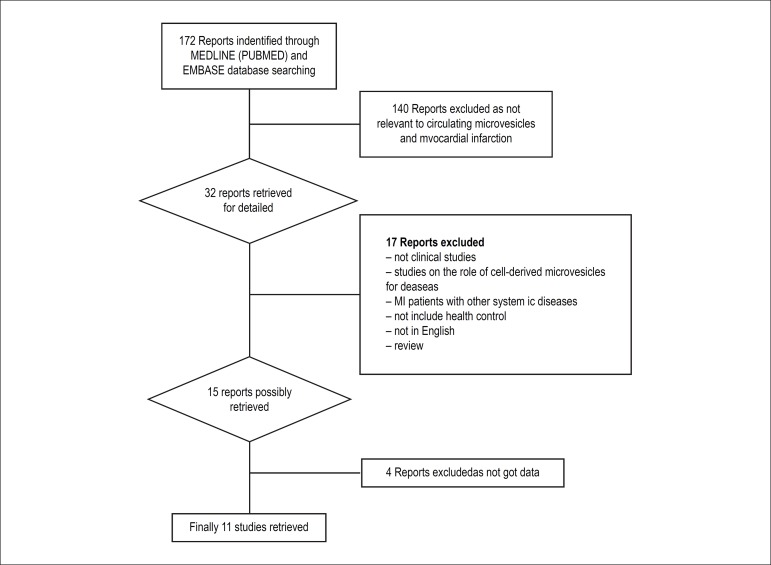
Flow diagram of search strategy and study selection.

172 articles identified from MEDLINE (pubmed), Embase, Cochrane and Google Scholar electronic database were analyzed; and then, 140 were excluded based on title and abstract. After detailed evaluation of potential eligibility, 11 studies met all the inclusion criteria and were retrieved for meta-analysis.^9-19^ The trial flowchart is summarized in Figure 1.

### Baseline Characteristics of the studies

The characteristics of all included studies are presented in Table 1. These studies were published from 2004 to 2013. Sample size ranged from 5 to 61. A total of 436 participants (186 healthy controls and 250 MI patients) were included. Among these studies, the results of MVs were expressed differently. Six reports were expressed as numbers of MVs in plasma per microliter, milliliter and liter. Three reports were expressed as PS eq (phosphatidylserine equivalents), one report was expressed as numbers of MVs in platelet count, and one report was expressed as plasma concentrations of MVs. There were four reports only report the median, range and the size of the trial. In order to estimate the mean and the variance in these articles, we used the number of sample and elementary inequalities.^20^

**Table 1:** Characteristic of included studies

Author/Year	Study object		Measurement method of MVs	Units of MVs	Sample size
	MI	Control			MI/Control
Cui Y/2013	STEMI/NSTEMI	Health	Flow cytometry	10^5^/mL	40/20
Del Turco S/2008	MI	Health	Flow cytometry	10^6^/L	46/10
Leong H S/2011	AMI	Health	Flow cytometry	/µL	6/5
Matsumoto N/2004	ACS	Health	Flow cytometry	104/platelet count	41/20
Michelsen A E/2008	MI	Health	BCA Protein Assay	µg/L	61/61
Min P K/2013	STEMI	Health	ELISA	nM (phosphatidylserine equivalent)	45/16
Morel O/2004	STEMI	Health	ELISA	nM (PhtdSer equivalent)	50/50
Morel O/2005	STEMI	Health	ELISA	nM (PhtdSer equivalent)	9/50
Skeppholm M/2012	STEMI/NSTEMI	Health	Flow cytometry	10^6^/L	51/61
Stepien E/2012	AMI	Health	Flow cytometry	/µL	12/9
Tan K T/2005	ACS	Health	Flow cytometry	10^5^/mL	54/35

### Quality Index

The majority of studies scored highly on reporting of the interventions used and outcome measures. Only one report scored lowly on the small sample size. The average score of all studies was 15.8 (Table 2).

**Table 2:** Quality Index of included studies

Author/Year	Quality Index
Cui Y/2013	16
Del Turco S/2008	16
Leong H S/2011	14
Matsumoto N/2004	16
Michelsen A E/2008	16
Min P K/2013	16
Morel O/2004	16
Morel O/2005	16
Skeppholm M/2012	16
Stepien E/2012	16
Tan K T/2005	16

### Annexin V+ microvesicles in health control and patients with myocardial infarction

**Figure 2:**
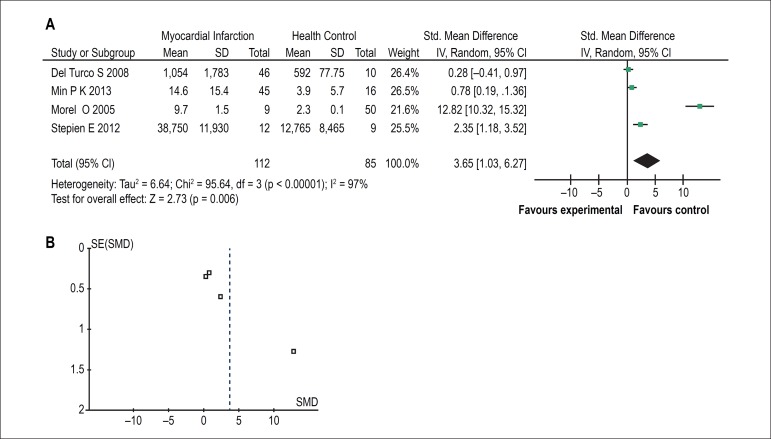
The forest plot (A) and funnel plot (B) of meta-analysis of Annexin V+ microvesicles in myocardial infarction.

Four of the eleven studies showed changes in AMVs levels between patients with myocardial infarction and healthy controls. In three of these reports, AMVs levels in patients with myocardial infarction were higher than healthy controls. Only in one study, patients with myocardial infarction did not differ from healthy controls concerning AMVs levels. When results of all studies were combined, there was a significant difference between groups with higher AMVs levels in patients with a myocardial infarction [SMD = 3.65, 95% CI (1.03, 6.27), Z = 2.73 (p < 0.00001; Figure 2A)]. Furthermore, there was significant statistical heterogeneity across studies (χ^2^= 95.64, df = 3, p < 0.00001, I^2^= 97%). As shown in Figure 2B, no publication bias was found.

### Platelet microvesicles in health control and patients with myocardial infarction

**Figure 3:**
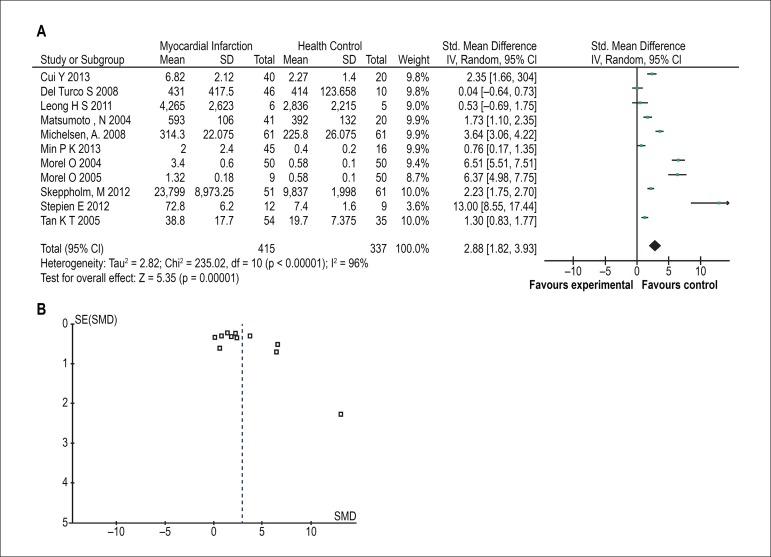
The forest plot (A) and funnel plot (B) meta-analysis of platelet microvesicles in myocardial infarction.

All of eleven studies found PMVs levels varied between myocardial infarction patients and healthy controls. Nine studies reported that level of PMVs were higher in MI patients, whereas the other studies showed no difference in groups. Combining the results of all studies, it was significantly increased in myocardial infarction patients [SMD = 2.88, 95% CI (1.82, 3.93), Z = 5.35 (p < 0.00001; Figure 3A)]. There was also significant statistical heterogeneity across studies (χ^2^= 235.02, df = 10, p < 0.00001, I^2^= 96%). As shown in Figure 3B, a little publication bias was found.

### Endothelial microvesicles in health control and patients with myocardial infarction

**Figure 4:**
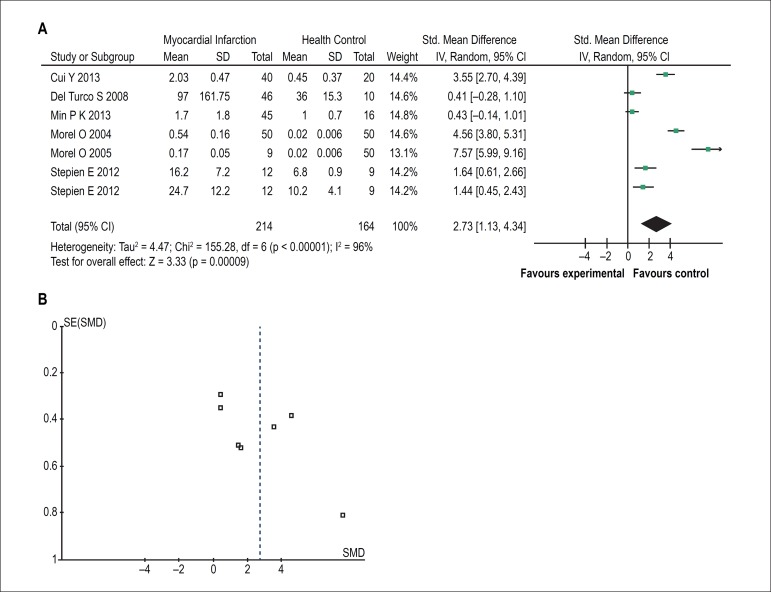
The forest plot (A) and funnel plot (B) meta-analysis of endothelial microvesicles in myocardial infarction.

Six of the eleven studies reported alternation in EMVs levels between the two groups. Four reports concluded that a proportion of MI patients have elevated EMVs levels. But the other studies showed no significant difference. Combining all results of those studies, MI patients had a higher level of EMVs. [SMD = 2.73, 95% CI (1.13, 4.34), Z = 3.33 (p = 0.0009; Figure 4A)]. The statistical heterogeneity was significant across studies (χ^2^= 155.28, df = 6, p < 0.00001, I^2^= 96%). As shown in Figure 4B, no publication bias was found.

### Leukocyte microvesicles in health control and patients with myocardial infarction

**Figure 5:**
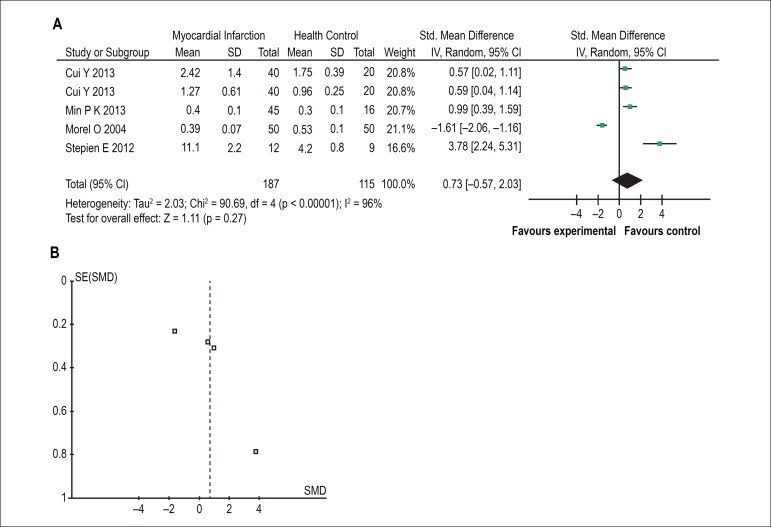
The forest plot (A) and funnel plot (B) meta-analysis of leukocyte microvesicles in myocardial infarction.

Five of the eleven studies exhibited differences in LMVs levels between myocardial infarction patients and health controls. Four reports showed that LMVs levels in myocardial infarction patients were higher than health controls. The finding of one report was, however, in the opposite direction, with patients having significantly lower LMVs levels than controls. When results of all studies were combined, there was no significant difference between the two groups [SMD = 0.73, 95% CI (-0.57, 2.03), Z = 1.11 (p = 0.27; Figure 5A)]. There was also significant statistical heterogeneity across studies (χ^2^= 90.69, df = 4, p < 0.00001, I^2^= 96%). As shown in Figure 5B, no publication bias was found.

## Discussion

We conducted an exhaustive search to identify studies related to our question and gave the most comprehensive overview of MVs in MI to date. Systematic methods were applied to reduce bias in the identification of studies, data extraction and synthesis, and appraisal of study quality. This meta-analysis showed that higher level of AMVs, PMVs and EMVs in peripheral blood might be associated with patients with MI, indicating that these MVs may be helpful in the diagnosis of MI. However, the result of LMVs was negative.

Microvesicles (MVS) or microparticles (MPS) have relationship not only with inflamatory and thrombotic processes but with tissue regenerative process and angiogenesis, which can be a protective function. In addition, MVS can be a signaler of homeostasis balancing cell stimulus and apoptosis. Diabetic patients have increased release of MVS and this can be a biomarker of diabetic progression by retinopathy. Pharmacologic approach is helpful because of endothelial dysfunction. Renin-angiotensin system blocker and calcium channel blocker may be good options in type 2 diabetes mellitus.^21^ Pinheiro et al.^22^ have assessed the effect of the antiplatelet drug clopidogrel in association or not with rosuvastatin (40 mg) on the levels of EMP and PMP in patients with stable coronary disease on statins for at least three months. Those authors have identified an increase in the levels of PMP after suspension of rosuvastatina and maintenance of only clopidogrel for four weeks and a tendency towards greater release of EMP in those patients. They have suggested that an increase in the apoptosis of platelets occurred, and that rosuvastatin might have a protective effect on the endothelium when associated with clopidogrel.^22^ In a similar study, França et al.^23^ have assessed the influence of atorvastatin (80 mg) in association or not with clopidogrel in patients with stable coronary disease. Those authors have suggested higher vascular stability promoted by atorvastatin after identifying an inverse relationship between the plasma concentration of atorvastatin and the levels of PMP.^23^

MVs have a bilayered phospholipid membrane.^24^ The presence of externalized PS on MVs surfaces indicates an altered phospholipid distribution profile compared to the plasma membrane of a resting mammalian cell. Additionally, the molecules present in the outer surface, once defined, provide accessible markers for detecting and characterizing MVs using molecular probes.^25^ AMVs express phosphatidylserine (PS) in their surface and are currently defined as apoptotic MVs. It is shown that AMVs levels were significantly higher in patients with MI than healthy controls. It is likely due to the myocardial ischemia and hypoxia which make cell apoptosis, thereby releasing large amounts of AMVs. In each case, the variation in AMVs levels shows that cell apoptosis occurs. A in vitro study uncovered that MVs extracted from the circulating blood of a patient with myocardial infarction and applied to the isolated aortic rings of a rat, led to severely damage of endothelial function.^26^ Thus, the high level of AMVs and their effect may be the cause the further progression of myocardial infarction.

PMVs are defined as membranous vesicles derived from the platelet which are defined and identified by surface molecules CD62P and CD63.^27^ With the ability to bind coagulation factors VIII, Va, and IX, PMVs not only reflect platelet activation but also contribute to the activation of the coagulation pathway and thrombogenesis.^28-29^ It was found that there was high level of PMVs in the peripheral blood of the MI patients. The main cause may be the change of blood flow shear force induced by pathological changes in the blood vessels of the MI patients. These changes promote the aggregation and activation of platelets, thereby leading to the generation of a large number of PMVs. This suggests that increased PMVs may be further expanded by the coagulation reaction of the blood vessels in the MI patients.

The dysfunction of endothelial cells plays an important role in the MI. EMVs are sub-cellular membrane vesicles released from endothelial cell during activation or apoptosis.^30^ EMVs carry specific markers which originate from the maternal cells, including CD31, CD51, CD54, CD62E, CD105, CD144 or CD146. It is found that EMVs were significantly higher in the MI group. His could be because of the dysfunction of endothelial cells, which release lots of EMVs into the blood. When the EMVs were incubated with human umbilical vein endothelial cells (HUVECs), endothelial cells proliferation decreased and their apoptosis increased, then the capacity of angiogenesis went down dramatically.^31^ The high level of EMVs is considered as a cause to worsen the condition by inducing endothelial dysfunction on the MI states. 

Unlike the single source of EMVs or PMVs, LMVs may originate from neutrophils, monocytes/macrophages, and lymphocytes.^32^ They also express markers from their parental cells. Various antibodies were used to capture LMVs, including CD4, CD14, CD11a and so on. Combined with the clinical results, this meta-analysis found that there was no significant variation in LMVs’ level between the health controls and patients with MI. But the studies that used CD4 and CD14 antibodies, shown LMVs’ level was higher in patients with MI. When CD11a antibody was used, a muddle of contradictory results was found. Therefore, additional studies are needed to further investigate the level of LMVs in MI. Meanwhile, LMVs measurement still requires elaborate techniques because of its lack of standardization.

Limitations of this meta-analysis must be considered. First, the quality of individual studies was not always optimal, as shown by the general lack of information on some studies. So we used simple and elementary inequalities in order to estimate the mean and the variance for such studies. But it is not exactly enough. Second, there is heterogeneity of SMD across studies, corresponding in part to heterogeneity in study definitions. Third, although the quality of included studies was judged overall to be adequate, the findings in the present study need to be interpreted with caution, given that not all studies reported on potential confounding variables and their adjustment in analyses. Finally, meta-analytic data also need to be cautiously interpreted, given the substantial heterogeneity among studies.

## Conclusion

This meta-analysis showed that higher level of AMVs, PMVs and EMVs in peripheral blood may be associated with patients with MI, indicating that these MVs may be helpful in the diagnosis of MI. However, the result of LMVs was negative.
